# Identification of QTLs Containing Resistance Genes for Sclerotinia Stem Rot in *Brassica napus* Using Comparative Transcriptomic Studies

**DOI:** 10.3389/fpls.2020.00776

**Published:** 2020-06-10

**Authors:** Muhammad Uzair Qasim, Qing Zhao, Muhammad Shahid, Rana Abdul Samad, Sunny Ahmar, Jian Wu, Chuchuan Fan, Yongming Zhou

**Affiliations:** ^1^National Key Laboratory of Crop Genetic Improvement, College of Plant Science and Technology, Huazhong Agricultural University, Wuhan, China; ^2^Jiangsu Provincial Key Laboratory of Crop Genetics and Physiology, Yangzhou University, Yangzhou, China

**Keywords:** Sclerotinia stem rot, quantitative trait loci, *Brassica napus* L., RNA sequencing, flowering time

## Abstract

Sclerotinia stem rot is a major disease in *Brassica napus* that causes yield losses of 10–20% and reaching 80% in severely infected fields. SSR not only causes yield reduction but also causes low oil quality by reducing fatty acid content. There is a need to identify resistant genetic sources with functional significance for the breeding of SSR-resistant cultivars. In this study, we identified 17 QTLs involved in SSR resistance in three different seasons using SNP markers and disease lesion development after artificial inoculation. There were no common QTLs in all 3 years, but there were three QTLs that appeared in two seasons covering all seasons with a shared QTL. The QTLs identified in the 2 years were *SRA9a*, *SRC2a* and *SRC3a* with phenotypic effect variances of 14.75 and 11.57% for *SRA9a*, 7.49 and 10.38% for *SRC3a* and 7.73 and 6.81% for *SRC2a* in their 2 years, respectively. The flowering time was also found to have a negative correlation with disease resistance, i.e., early-maturing lines were more susceptible to disease. The stem width has shown a notably weak effect on disease development, causing researchers to ignore its effect. Given that flowering time is an important factor in disease resistance, we used comparative RNA-sequencing analysis of resistant and susceptible lines with consistent performance in 3 years with almost the same flowering time to identify the resistance genes directly involved in resistance within the QTL regions. Overall, there were more genes differentially expressed in resistant lines 19,970 than in susceptible lines 3936 compared to their mock-inoculated lines, demonstrating their tendency to cope with disease. We identified 36 putative candidate genes from the resistant lines that were upregulated in resistant lines compared to resistant mock and susceptible lines that might be involved in resistance to SSR.

## Introduction

*Sclerotinia sclerotiorum* (Lib.) de Bary is a necrotrophic and non-host-specific fungal pathogen that infects more than 400 plant species, including several valuable oil crops, such as oilseed rape, soybean, and sunflower (Purdy, 1979; Boland and Hall, 1994). *S. sclerotiorum* is a one of the major disease of rapeseed (*Brassica napus*), causing yield losses from 10–20% and up to 80% with severe attack (Mei et al., 2011). The disease also costs low oil content and affect the quality of oil by changing fatty acid profile (McCartney et al., 1999).

*Sclerotinia sclerotiorum* is difficult to manage via cultural practices because it has a wide host variety and a long-lived melanized resting structure. The control of the disease is possible with fungicide application if applied properly at the optimum time. However, identifying the optimum time is a difficult task, and it is a costly method that also adds to environmental pollution. Thus, developing and cultivating resistant varieties is necessary to cope with this disease. Many techniques have been developed to identify the genes, genetic networks, and genome regions contributing for the traits. Some of these techniques have been applied for the identification of resistant genetic resources against Sclerotinia stem rot (SSR) in *Brassica napus.* Some of the candidate resistance genes against *S. sclerotiorum* have been identified via quantitative trait loci (QTL) mapping, genome-wide association studies (GWAS), RNA-sequencing (RNA-seq) and other techniques (Mei et al., 2010, 2012; Wei et al., 2014, 2016; Wu et al., 2013, 2016a, 2016b, 2019). Two segregating populations developed from partial resistant and susceptible cultivars revealed ten QTLs on six chromosomes with a phenotypic variance effect (*R*^2^) of 6-21% (Zhao et al., 2006). A candidate resistance gene has been identified on chromosome C6 in *B. napus*, contributing a *R*^2^ value of 29.01–32.61% in 3 years at two locations (Wu et al., 2013). Most of the resistant QTLs identified in *B. napus* to date are contributed by the *Brassica olerecea* genome, and six QTLs characterized in another study for SSR resistance are contributed by the *B. olerecea* genome with *R*^2^ 2.2 to 24.8% in 2 years (Mei et al., 2013). GWAS has also been employed to identify loci involved in resistance against SSR, and 26 SNPs associated with disease resistance have been identified on chromosomes C4, C6, and C8 with *R*^2^ of 6.14, 5.08, and 5.26%, respectively. Combining previous work on differential gene expression with GWAS, these researchers also predicted 39 genes to be involved in SSR resistance (Wu et al., 2016a). In another genome-wide association study along with differential gene expression, 17 significant SNP associations with SSR resistance were identified on chromosomes A8 and C6, and 24 genes for resistance were identified by combining SNP association analysis and transcriptomic studies (Wei et al., 2016). Comparative transcriptomic studies revealed a network of genes involved in resistance to SSR when differential gene expression was studied (Wu et al., 2016b). Despite all these efforts to identify sources of resistance, there is still a gap for having a firm genetic source of resistance against SSR, and more studies are needed to achieve complete genetic control over the disease.

In *B. napus*, SSR resistance is a combined effect of many QTLs with little contribution to the trait (Wu et al., 2016a). Other than genetic resources, which directly influence the disease, there may be other factors that may influence disease resistance e.g., flowering time is an important developmental stage that may have a role in plant pathogen interactions (Kazan and Lyons, 2016). The colocalization of QTLs responsible for SSR resistance and flowering time has been identified earlier (Wu et al., 2019). A negative correlation between flowering time and SSR resistance was identified in another study with some common QTL regions for both traits (Wei et al., 2014). In addition to flowering time, stem width may have an effect on disease occurrence, especially when artificially induced. Considering plant development, we attempted to maintain an equal number of plants in each plot. In this study, with our main focus on the identification of QTLs and genes within QTL regions for SSR resistance, we also attempted to cover two plant traits that may affect resistance, i.e., flowering time and stem width. We identified 17 QTLs in three consecutive years and found that early flowering plants are more susceptible to disease and that stem width has a notably weak effect on disease response. We focused on identifying resistance genes that are directly involved in resistance due to genetic makeup, rather than plant growth achievement. We conducted comparative RNA-seq analysis to identify the genes differentially expressed in resistant and susceptible lines within QTL regions with almost the same flowering time from the DH population to identify the genes directly involved in SSR resistance.

## Materials and Methods

### Plant Material

Two extreme parent lines with resistance J964 and susceptibility J902 were used to develop the double haploid (DH) population for this study. The cross was made between two parents, and F_1_ was developed. A DH population was developed using anther culture of the F_1_ population. A total of 181 DH lines were developed using anther culture and were used for trait analysis and QTL mapping. All materials were grown and phenotypic measurement were recorded on the experimental farm of Huazhong Agriculture University, Wuhan, China, in the winter-type oilseed rape growing season in 2015–2016, 2016–2017, 2017–2018 and for measuring flowering time of DH lines used for RNA-seq grown again in 2018–2019. Parents along with the DH lines were grown in three replications following randomized complete block design (RCBD). The distance between the plants was kept 21 cm in each row and 30 cm between the rows. The field was managed following normal breeding practices.

### Phenotyping Measurements and Statistical Analysis

Parents along with DH lines were artificially inoculated with *S. sclerotiorum* for three consecutive years. A mycelial agar plug (7 mm in diameter) punched from the margin of a 2-day-old culture of *S. sclerotiorum* grown on potato dextrose agar (PDA) was used as the inoculum. Lesion length was noted in centimeters after 7 days of inoculation.

Flowering data were noted in 2017 for three replications of the DH population. When the 25% of plants in each replication of each genotype started flowering, it was noted as initial flowering. There was only a small gap in days from initial (25%) to final (100%) flowering, as most of the plants from the same lines start flowering at the same time. The initial flowering time was then used for the correlation between flowering time and lesion length. Flowering time was also noted in 2019 for selected plants that were later used for RNA-seq analysis. Stem width data were taken in 2018 from 30 random genotypes, including three replications for each genotype. The stem width (diameter) was measured using a Vernier caliper in millimeters from the same internode and slightly above the point where inoculant was applied. Regression analysis (simple linear regression model) was performed using Microsoft Excel with lesion length (as the dependent variable) to flowering time (as the independent variable) and was the same for plant width, with width replacing flowering time.

### SNP Array Genotyping

Genomic DNA was extracted from 181 DH lines and parents grown in 2016 using the CTAB method as described earlier (Murray and Thompson, 1980). A total of 181 genotypes selected were used for genotyping along with parents. Genotyping was performed using Brassica 6 K Illumina Infinium HD Assay SNP arrays (Illumina Inc., San Diego, CA, United States) according to the manufacturer’s protocol (Infinium HD Assay Ultra Protocol Guide^1^) (Mason et al., 2017). Illumina GenomeStudio software (Illumina Inc., San Diego, CA, United States) was used to cluster and visualize all SNP array data (Supplementary Table S1) for further analysis. Each SNP was rechecked manually to see if any error was there during the clustering analysis.

### Linkage Mapping and QTL Map Construction

The SNP array data processing for the DH population followed a bi-filtering method described by Cai et al. (2015) was used, and the markers with same genotypes for the DH population were considered redundant SNPs and were removed. We only used the non-redundant SNPs (nrSNPs) for linkage map construction with MAPMAKER/EXP 3.0 and MSTmap software as described by Cai et al. (2014). The genetic distances between the markers was calculated using kosambi mapping function. QTL analysis was conducted using composite interval mapping (CIM) (Zeng, 1994) with Windows version of QTL cartographer 2.5 software^2^ with the same protocol described earlier (Wu et al., 2013). A significance threshold (*P* = 0.05) for QTL was determined through permutation analysis using 1000 repetitions. Forward and reverse regression analysis was applied for QTL detection. The confidence interval of QTL was measured at 1-LOD intervals surrounding the QTL peak.

### Sampling for RNA-seq Analysis

Comparative RNA-seq was performed for the two lines, one resistant and the other susceptible, with a consistent pattern of lesions in all 3 years of artificial inoculation. The main stems of the plants were inoculated at three sites on the neighboring internodes 40–60 cm above the ground using mycelial agar plugs. Mock-inoculated plants were inoculated with agar plugs only without any fungal growth. Epidermal stem tissues were harvested from 10 mm beyond the inoculation site and 1 mm deep at 24, 48, and 96 hours post-inoculation (hpi). Samples from each plant were mixed together from all three sites and considered as one replication, and three plants were used for three replications at one time point from each line for disease inoculation. One plant was mock-inoculated in each line at each time point, making three replicates covering three time points for mock-inoculated resistant and susceptible lines. The sampled tissues were immediately frozen in liquid nitrogen and later stored at −80°C before use for RNA extraction.

### RNA Quantification and Qualification, cDNA Library Construction and Sequencing

RNA was extracted from all 24 samples by using an NEB extraction kit following protocols by the manufacturers. RNA degradation and contamination were checked on 1% agarose gels. RNA purity was tested using a NanoPhotometer^®^ spectrophotometer (IMPLEN, CA, United States). RNA integrity was assessed using the RNA Nano 6000 Assay Kit of the Bioanalyzer 2100 system (Agilent Technologies, CA, United States). Sequencing libraries were created using the NEBNext^®^ UltraTM RNA Library Prep Kit for Illumina^®^ (NEB, United States) following the manufacturer’s recommendations, and index codes were added to attribute sequences to each sample. The clustering of the index-coded samples was performed on a cBot Cluster Generation System using TruSeq PE Cluster Kit v3-cBot-HS (Illumina) according to the manufacturer’s instructions. After cluster generation, the library preparations were sequenced on an Illumina Novaseq platform, and 150-bp paired-end reads were produced.

### Quality Control and Read Mapping to the Reference Genome

Raw data (raw reads) in fastq format were first processed through in-house Perl scripts. In this step, clean data (clean reads) were obtained by removing reads containing adapters, reads containing poly-N and low-quality reads from raw data. At the same time, Q20, Q30 and GC content of the clean data were calculated. All downstream analyses were conducted with clean data with high quality. ‘Darmor-bzh’ reference genome and gene model annotation files (Chalhoub et al., 2014) were downloaded from the genome website. The index of the reference genome was built using Hisat2 v2.0.5, and paired-end clean reads were aligned to the reference genome using Hisat2 v2.0.5^3^. We selected Hisat2 as the mapping tool because Hisat2 can generate a database of splice junctions based on the gene model annotation file and thus a better mapping result than other non-splice mapping tools.

### Differential Gene Expression Quantification

Feature Counts v1.5.0-p3 (Liao et al., 2014) was used to count the read numbers mapped to each gene, and then fragments per kilobase of transcript sequence per million (FPKM) fragments mapped of each gene was calculated based on the length of the gene and read count mapped to this gene. The FPKM considers the effect of sequencing depth and gene length for the read count at the same time and is currently the most commonly used method for estimating gene expression levels. Differential expression analysis of two groups (including biological replicates per group) was performed using the DESeq2 R package (1.16.1) (Love et al., 2014). DESeq2 provides statistical routines for measuring differential expression in digital gene expression data using a model based on the negative binomial distribution. The resulting *P*-values were adjusted using Benjamini and Hochberg’s method (Haynes, 2013) for monitoring the false discovery rate. Genes with an adjusted *P*-value < 0.05 found by DESeq2 were considered as DEGs.

### qPCR Analysis for the Validation of RNA-seq Data

The expression pattern of the genes obtained from RNA-seq analysis was validated by performing the Quantitative real-time polymerase chain reaction (qRT-PCR). The expression pattern of the nine selected genes was studied with qRT-PCR analysis using the same samples used for RNA-seq analysis. The qRT-PCR assay was carried out as explained earlier by Shahid et al. (2019) using three biological replicates repeated twice as technical replicates. SYBR-Green MasterMix was used to perform qRT-PCR experiment. The gene copy specific primers were designed using Premier-5 program. The relative cycle threshold (Ct) value was used to measure relative expression by the 2^–Δ^ ^Ct^ method. *BnaA09g14410D* (PP2A-1) gene was used as control to normalize the expression data (Zhao et al., 2019). The detail of genes and primers are listed in Supplementary Table S4.

## Results

### Phenotype of the DH Lines

**FIGURE 1:**
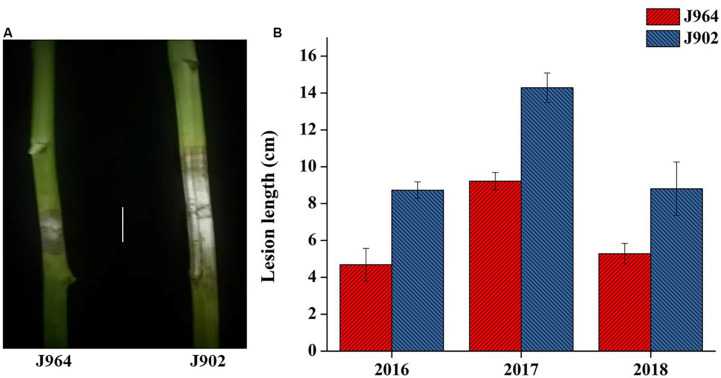
Phenotype of parents. **(A)** Picture of the lesion length on plants stemming from J964 resistant and J902 susceptible parent plants in 2018 (Scale bar: 4 cm). **(B)** Lesion length of parent J964 resistant and J902 susceptible plants taken in 3 years.

**FIGURE 2:**
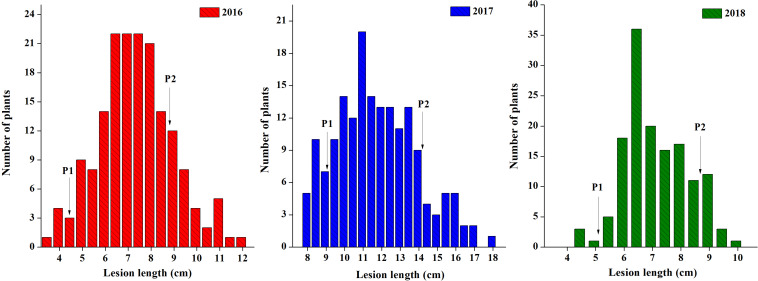
Frequency distribution of lesion length for the DH population in 3 years. The arrows indicate the mean lesion length of parent lines. P1 is J964 the resistant parent, and P2 is J902 the susceptible parent.

Parent lines showed clear differences in lesion length, noted 7 days post-inoculation (dpi) in all 3 years (Figure 1). The DH population developed from F_1_ of two extreme parents assayed after 7 dpi revealed continuous segregation. It can be observed from the lesion length distribution that this trait is controlled quantitatively. Disease lesion length distribution among the population recorded at 7 dpi for the 3 years ranged from 3.43 to 17.80 cm in extreme lines. The frequency distribution of lesion length in 3 years among the DH population is shown in Figure 2.

The correlation among the 3 years of data was noted to see the consistency of the resistance pattern in DH lines. The correlation was highly significant, indicating the same pattern of lesion development among the genotypes and confirming the credibility of the method used (Table 1). The heritability for SSR resistance is as high as 68.36% (Table 2), indicating that the phenotype is primarily attributable to genetic factors with less environmental effects.

**TABLE 1:** Correlation among 3-year lesion length of the DH population.

	**2016**	**2017**	**2018**
2016	1		
2017	0.67**	1	
2018	0.51**	0.44**	1

** indicates the significance at *p* < 0.01.

**TABLE 2:** Two-way ANOVA and broad sense heritability (*h*^2^) of SSR resistance in the DH population.

**Source**	**df**	**SS**	**MS**	***F***	**Sig**
Season	2	8495.46	4247.73	955.60	**
Rep	2	880.41	440.20	99.03	**
Genotype	287	4032.74	14.05	3.16	**
Season x genotype	459	2644.46	5.77	1.30	**
Error	1494	6640.96	4.45		
Total	2245	192055.09			
			Heritability	0.68	68.36%

df, degree of freedom. SS, sum of squares. MS, mean square. ** indicates the significance at *p* < 0.01.

#### Correlation Among Stem Width and Lesion Length

**FIGURE 3:**
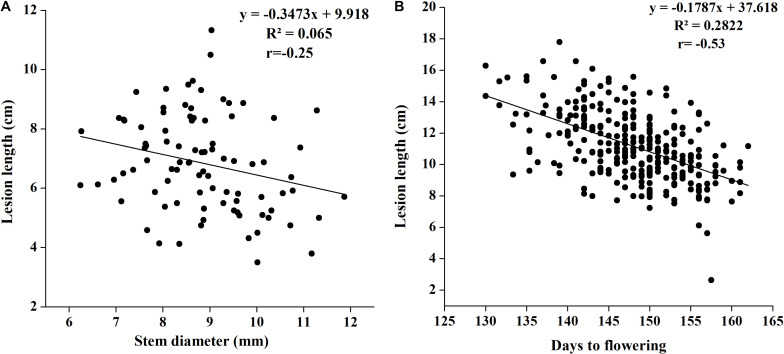
**(A)** Regression analysis of SSR resistance and stem width in 2018. **(B)** Regression analysis of SSR resistance and flowering time in 2017.

Although we attempted to maintain an equal number of plants in every row, plant development showed slight differences in such parameters as stem width. To check whether stem width affects disease lesion development, we noted plant stem diameter in 2018 with three replications and correlated it with lesion length. We found a weak negative correlation between stem width and lesion length, and the coefficient of correlation was low at −0.2552 (*p* = 0.0152). The regression of stem width on lesion length was also notably low (0.065), indicating the important role played by plant genetic makeup in disease resistance, rather than its growth achievement (Figure 3A).

### Correlation Between Flowering Time and Disease Resistance

There are evidences that flowering time in *B. napus* affects SSR resistance (Wei et al., 2014; Wu et al., 2019); therefore, we noted differences in flowering initiation and flowering time in our study to determine whether flowering time affects disease resistance. Flowering time was found to be significantly negatively associated with disease lesion length (Figure 3B). The coefficient of correlation was as high as −0.53, suggesting that the plants with early flowering have greater susceptibility to disease with increased lesion length.

### QTL Mapping

Using the 1401 SNP polymorphic markers for the parents and DH population of 181 lines, a linkage map was constructed that covered a distance of 972 cM with the kosambi function (Table 3). Seventeen QTLs were identified in three seasons using a linkage map and phenotype data of 3 years for disease lesion length. All 17 identified QTLs are present on six chromosomes out of 19 chromosomes in *B. napus*.

**TABLE 3:** Linkage map of all the chromosomes and their summary.

**Chromosome**	**Length (cM)**	**Number of Markers**	**Ave. distance (cM)**	**Min (bp)**	**Max (bp)**
A01	48.5	54	0.90	198823	22994071
A02	48.7	66	0.74	695308	23281113
A03	60.5	66	0.92	789737	26015888
A04	27.7	97	0.29	104399	16546328
A05	51.3	68	0.75	1091785	22464190
A06	59.2	108	0.55	58478	24366029
A07	33.1	64	0.52	963624	20046365
A08	35.5	58	0.61	636677	18068968
A09	70.7	60	1.18	2261544	33470049
A10	52.7	69	0.76	885133	17271592
C01	51.3	45	1.14	990251	36483867
C02	49	120	0.41	4456183	46038200
C03	86.6	120	0.72	2433	60450966
C04	58.1	72	0.81	661	46986106
C05	67.4	61	1.10	328062	42644430
C06	54.9	122	0.45	3555794	35254379
C07	42.6	74	0.58	22182153	44303399
C08	37.1	70	0.53	1345885	32127235
C09	37.8	7	5.40	13196099	45218259
A_Subgenome	487.9	710			
C_Subgenome	484.8	691			
Total	972.7	1401			

*Length, length of linkage map in centimorgans. Marker, total number of markers used. Ave. distance, average distance of markers within chromosome. Min, start position of markers within chromosomes. Max, end position of markers within chromosomes.*

Seven QTLs were identified in 2016 on chromosomes A2, A9, and C3, five QTLs in 2017 on chromosomes A2, C2, C3, and C4, and five QTLs in 2018 on chromosomes A9, C2, and C6 (Table 4). None of the QTLs was identified in all 3 years. The chromosomes having QTLs for 2 years were A9 (2016, 2018), A2 (2016, 2017), C2 (2017, 2018), and C3 (2016, 2017). Among these QTLs identified, *SRA9a* in 2016 lies between the confidence interval for *SRA9a* in 2018 with *R*^2^ 14 and 11%, respectively. *SRC3a* for 2016 lies within the confidence interval of *SRC3a* in 2017 with *R*^2^ 7 and 10%, respectively. *SRC2a* in 2017 also lies within the confidence interval of QTL *SRC2a* for 2018, with *R*^2^ 7.73 and 6.81%, respectively. All of the pairs of common QTLs with overlapping confidence intervals showed additive effects from the same common parent. These overlapping QTLs in 2 years are assigned the same name. It can be concluded that the same genes may be involved in resistance in different growing seasons, which warrants further research.

**TABLE 4:** QTLs identified in the DH population in three seasons.

**Season**	**QTL**	**Chr**	**Peak**	**LOD**	**Interval (cM)**	**Additive**	***R*^2^ (%)**
2016	*SRA2a*	A2	25.71	3.47	23.6–29	−0.4993	6.96
	*SRA9a*	A9	85.91	4.22	76.4–96.9	−0.6483	14.75
	*SRA9c*	A9	100.51	3.97	97.6–104.6	−0.469	7.62
	*SRC3a*	C3	21.11	3.43	13.6–28.7	0.7304	7.49
	*SRC3b*	C3	78.01	3.04	75.2–81.2	0.4126	6.20
	*SRC3c*	C3	84.71	3.54	81.2–89.4	0.4418	7.16
	*SRC3d*	C3	90.01	2.92	89.4–96.4	0.3996	5.94
2017	*SRA2b*	A2	21.21	5.26	18.5–21.9	−0.7687	10.74
	*SRA2c*	A2	31.31	6.12	29–36.4	−0.9078	14.27
	*SRC2a*	C2a	5.51	3.88	1.6–14.8	−0.8287	7.73
	*SRC3a*	C3	17.81	3.75	7.3–30.5	1.0179	10.38
	*SRC4*	C4	34.31	4.87	32–40	−0.7326	9.82
2018	*SRA9b*	A9	69.31	2.72	67.9–72.3	−0.3084	6.39
	*SRA9a*	A9	82.91	3.04	74.5–96.9	−0.4045	11.57
	*SRC2a*	C2a	9.61	2.52	0–17.3	−0.3	6.81
	*SRC2b*	C2b	35.11	2.95	30.1–41.3	−0.3495	9.61
	*SRC6*	C6	49.11	3.35	43.9–53.6	−0.3479	8.75

*QTL nomenclature uses the abbreviation of the trait followed by the chromosome number; an alphabetical letter a or b or c is added if more than one QTL is identified in one chromosome. Chr, chromosome. Peak map position (cM) of peak. LOD (likelihood of odds) scores. Intervals (confidence intervals) of the QTLs identified. Additive effect: positive additivity indicates that the QTL allele originating from the parental line P1 (resistant) increases disease lesion length; negative additivity indicates that the QTL alleles originating from the susceptible parent line P2 increase disease lesion length. R2 proportion for the phenotypic variation explained by the QTL.*

### Integration of QTLs for SSR Resistance

**FIGURE 4:**
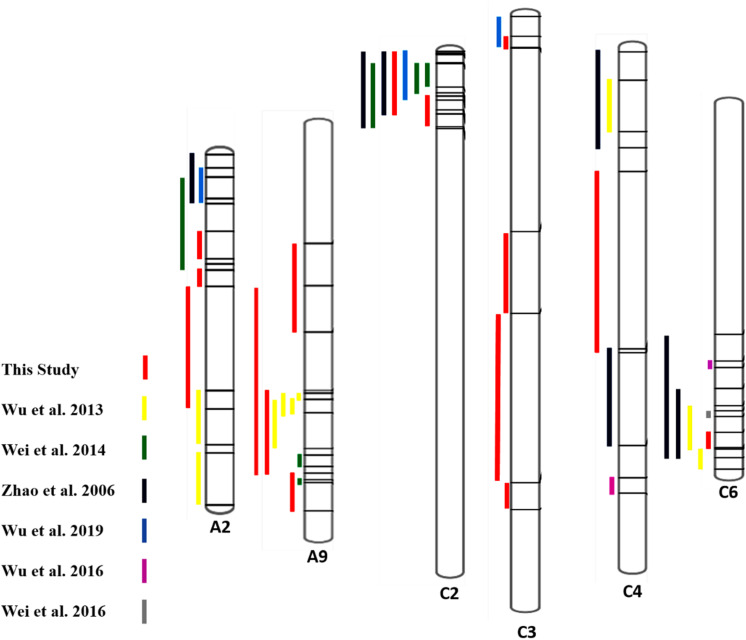
Comparison of QTLs identified for SSR resistance in the present and previous studies (Zhao et al., 2006; Wu et al., 2013, 2016a, 2019; Wei et al., 2014, 2016) based on available marker positions, including peaks.

For more confirmation about the QTL regions possessing the genes contributing to SSR resistance, we collected some evidence from some past studies and compared them with our findings (Figure 4). We collected information on QTLs and their physical positions in the linkage group based on marker information from past studies and compared it with our findings (Supplementary Table S2). The overlapping regions among the QTLs identified in this study and some previous studies were observed, and 4 out of six chromosomes having QTLs from this study showed overlapping regions with past studies. QTL *SRC2a* on chromosome C2 identified in two seasons (2017 and 2018) in the present study have shown overlapping confidence intervals with the QTLs identified by Zhao et al. (2006); Wei et al. (2014), and Wu et al. (2019). QTL *SRC3a* sharing overlapping confidence intervals in this study for two seasons also shows overlapping confidence intervals with the previous finding of Wu et al. (2019). The QTL *SRA9a*, which is also present in two seasons, has shown overlapping confidence intervals with QTLs identified by Wu et al. (2013). The QTLs identified only in one season on A9 *SRA9b* have an overlapping confidence interval with the QTL identified by Wei et al. (2014), and *SRA2c* has an overlapping confidence interval with Wu et al. (2013). The QTL *SRC6* identified on chromosome C6 in 2018 showed an overlapping confidence interval with Zhao et al. (2006) and Wu et al. (2013). To identify the genes responsible for resistance to SSR, we conducted RNA-seq analysis.

Also, the QTLs identified in other studies were on chromosomes A01, A03, A06, A08, A10, C03, C08, and C09 (Zhao et al., 2006; Wu et al., 2013, 2016a, 2019; Wei et al., 2014, 2016).

### Phenotype of the Lines Used for RNA-seq Analysis

**FIGURE 5:**
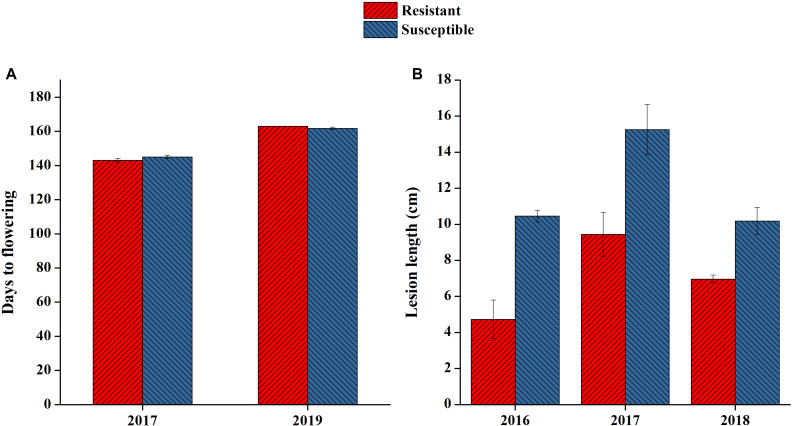
Phenotypic performance of resistant and susceptible lines selected for RNA sequencing. **(A)** Flowering time of the two lines selected in 2 years. **(B)** Lesion length of lines selected in 3 years.

On the basis of 3 years of lesion length data, two consistent lines of resistant and susceptible genotypes were selected for RNA-seq analysis. Both lines had clear differences in their lesion length patterns in all 3-year extremes ranging from 4.7 to 15.2 (Figure 5B). The lines selected also had similar flowering times, as we noted that flowering time also affected plant disease resistance patterns (Figure 5A). In this study, we only attempted to focus on the mechanisms directly affecting SSR resistance in *B. napus*, as both lines were at the same growth period when artificially inoculated.

### RNA-seq and Differential Gene Expression

All 24 samples subjected to paired-end RNA-seq revealed 1148.54 million clean reads with an average of 47.85 million clean reads in each sample (Supplementary Table S3). The fastq files of raw data are submitted at Sequence Read Archive database (SRA accession PRJNA616079). An average of 40.82 million reads were mapped on the *Brassica napus* genome, and on average, 80% of the reads were uniquely mapped in all samples. Considering FPKM thresh hold value of more than 1 in all three replications of resistant lines inoculated with PDA without fungus (R-mock) and susceptible lines inoculated with PDA without fungus (S-mock), 40,491 and 39,104 genes were expressed, respectively. Keeping the values ranging from 0.1 to 0.9 in every replication 2237 and 1891 genes were expressed in R-mock plants and S-mock plants, respectively, this low expression might be due to the stage-specific or tissue-specific nature of the genes. The Pearson correlation coefficient between the replications in both resistant lines and susceptible lines was calculated as high as 0.9 in most of the cases (Supplementary Figure 1), indicating the credibility of the RNA-seq method and samples. RNA-seq data were also validated with qPCR analysis with nine genes selected for qPCR, The Pearson correlation was calculated among FPKM values of RNA-seq data of same genes and qPCR relative expression for the resistant lines and was found to be higher as 0.99, assuring the reliability of RNA-seq analysis (Supplementary Table S4).

**FIGURE 6:**
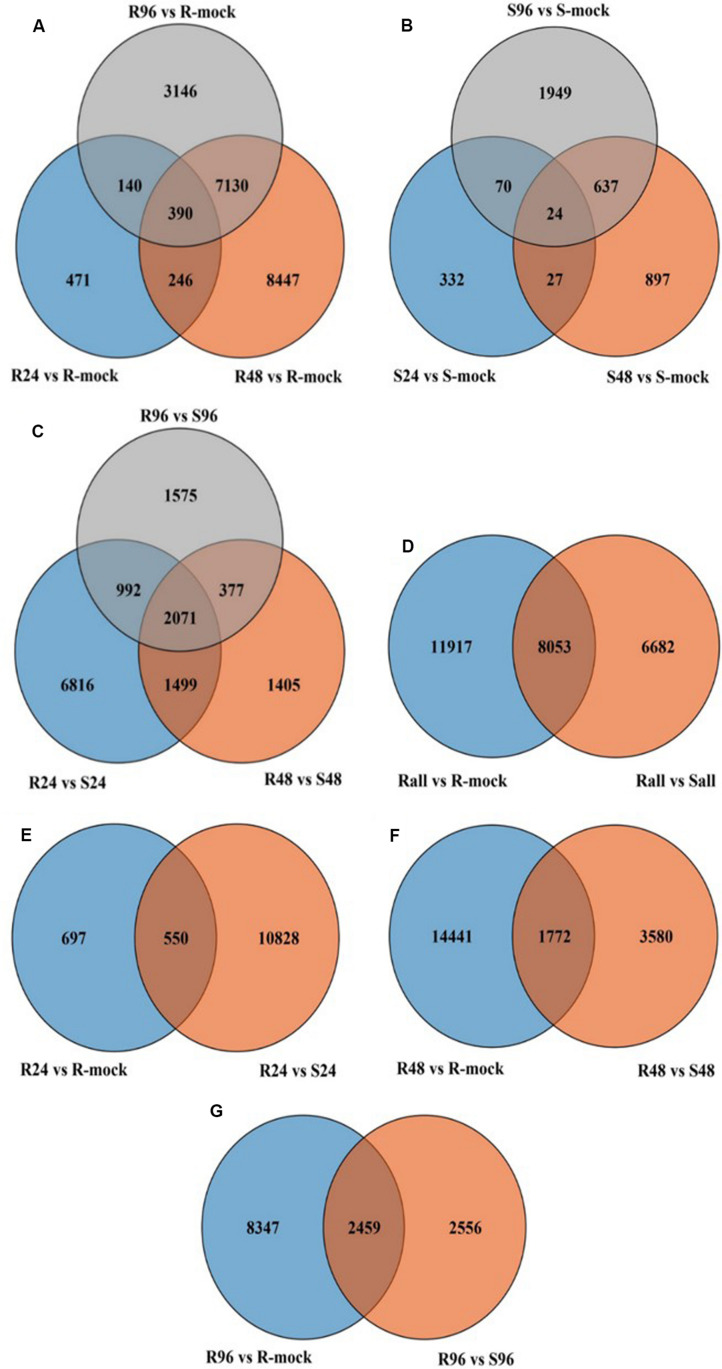
Venn diagram of different comparisons. **(A)** All genes in R vs. R-mock at all-time points. **(B)** All genes in S vs. S-mock at all time points. **(C)** All genes in R vs. S at all time points. **(D)** All genes in R vs. R-mock and R vs. S at all time points. **(E–G)** All genes at different time point comparisons between R vs. R-mock and R vs. S.

In resistant lines inoculated with disease (R) compared to R-mock lines, more genes were differentially expressed than in susceptible lines inoculated with disease (S) compared to S-mock lines. In total, 19,970 differentially expressed genes (DEGs) were found in R lines compared to R-mock lines (Figure 6A), and 3936 DEGs were found in S lines compared to S-mock lines at all time points (Figure 6B). Notably, after disease inoculation, there were a large number of DEGs in resistant lines compared to its mock line than in susceptible lines compared to its mock line. We also compared the DEGs between the R and S lines, and there were 14,735 genes in total at all three time points, including time-specific or DEGs sharing different time points in response to disease (Figure 6C). The large number of DEGs in R lines compared to S lines in response to disease inoculation indicates the tendency of resistant lines to cope with the disease.

### Identification of Putative Candidate Genes

Among RNA-seq for differentially expressed genes, we searched for the genes differentially expressed in our QTL regions, as these QTLs are already identified to be the regions containing genes affecting the disease resistance phenotype.

We compared R lines versus R-mock lines and R lines versus S lines, and there were more DEGs in the R vs. R-mock comparison compared to the R vs. S comparison. A total of 11,917 DEGs were there in the R vs. R-mock comparison, 6682 DEGs in the R vs. S comparison, and 8053 genes were expressed mutually in both comparisons at all time points (Figure 6D). We searched for the DEGs common in the R vs. R-mock and R vs. S comparisons at the same time point only (Figures 6E–G), and there were 550, 1172, and 2459 DEGs at 24, 48, and 96 hpi, respectively. In this study, we focused on the QTL positions identified in 2 years with the same confidence interval for DEGs. These QTLs were also identified in past studies as explained earlier. For the QTL region, there were 253 DEGs for *SRA9a*, 89 for *SRC2a* and 91 for *SRC3a* between R versus R-mock and 154 DEGs for *SRA9a*, 214 for *SRC2a* and 60 for *SRC3a* between R versus S at all three time points (Supplementary Table S5). Considering the genes only upregulated in one comparison, i.e., R vs. R-mock or R vs. S, may not contribute enough to the disease resistance trait. As the DEGs only in R vs. R-mock and not DEGs in comparison to S lines might not contribute enough to resistance and the DEGs only in R lines compared to S lines might be other varietal differences and not in response to disease. In common, 14 upregulated genes were found in *SRA9a*, 14 in *SRC2a* and 8 genes in *SRC3a* between both comparisons at different time points (Table 5). Most of the upregulated genes identified in QTL regions that were differentially expressed in both comparisons had been reported in previous studies to be involved in biotic or abiotic stresses. We included these 36 genes from three QTLs as putative candidate genes for SSR resistance.

**TABLE 5:** Genes from the QTL regions identified using differential gene expression from RNA-seq data.

**GENE_ID**	**R24 vs. S24**	**R24 vs. RM**	**R48 vs. S48**	**R48 vs. RM**	**R96 vs. S96**	**R96 vs. RM**	**Description**
BnaC03g05380D	9.69	–	10.56	1.17	10.24	–	Disease resistance protein (TIR-NBS-LRR class)
BnaC03g06010D	7.82	2.34	2.01	2.49	2.33	2.79	Zinc induced facilitator 1
BnaC03g06020D	10.31	–	6.96	3.81	7.41	–	Zinc induced facilitator-like 1
BnaC03g05390D	1.38	–	1.49	1.36	–	–	Las1-like family protein
BnaC03g04980D	–	–	0.81	–	1.33	1.50	Glycosyl hydrolases family 31 protein
BnaC03g06750D	–	–	–	2.86	1.05	3.33	Protein of unknown function (DUF1637)
BnaC03g04830D	0.49	–	–	–	0.84	1.63	Aspartate aminotransferase 3
BnaC03g06720D	1.34	2.52	–	–	1.65	3.13	Phosphoglycerate mutase-like family protein
BnaC02g09040D	3.87	–	7.60	3.20	6.55	–	ARM repeat superfamily protein
BnaC02g06950D	–	–	4.09	4.84	–	–	AAA-type ATPase family protein
BnaC02g06410D	4.76	–	6.81	1.34	6.55	–	Alpha/beta-Hydrolases superfamily protein
BnaC02g09520D	3.57	–	5.14	1.81	3.61	–	Alpha/beta-Hydrolases superfamily protein
BnaC02g09450D	3.71	0.93	3.64	2.25	4.23	2.71	Nitrilase 4
BnaC02g45360D	7.91	–	9.10	6.11	7.14	6.88	*S*-adenosyl-L-methionine-dependent methyltransferases superfamily protein
BnaC02g45710D	2.45	0.92	3.01	–	–	−2.12	HCP-like superfamily protein with MYND-type zinc finger
BnaC02g48820D	6.27	–	6.05	4.31	8.88	5.50	EXORDIUM like 2
BnaC02g09720D	8.03	–	7.85	2.18	5.80	–	Histone deacetylase 2B
BnaC02g09390D	12.29	–	8.70	1.98	8.34	1.97	Polynucleotidyl transferase, ribonuclease H-like superfamily protein
BnaC02g00990D	10.34	1.82	9.93	2.99	7.47	2.82	NAC (No Apical Meristem) domain transcriptional regulator superfamily protein
BnaC02g45750D	–	–	2.66	2.04	–	–	ELMO/CED-12 family protein
BnaC02g03430D	2.18	–	2.10	1.15	1.58	–	Ribosomal protein S12/S23 family protein
BnaC02g02880D	6.02	–	6.37	1.28	5.34	–	Ribosomal protein S28
BnaA09g36770D	1.32	1.12	–	2.16	–	2.16	Insulinase (Peptidase family M16) family protein
BnaA09g40500D	–	–	2.35	8.48	–	8.53	1-phosphatidylinositol-4-phosphate 5-kinase 3
BnaA09g40730D	0.54	–	1.01	0.72	–	–	KH domain-containing protein
BnaA09g30480D	–	–	1.35	2.46	–	2.94	Plant neutral invertase family protein
BnaA09g33340D	–	–	1.14	1.10	1.04	2.01	Pyruvate kinase family protein
BnaA09g33440D	8.05	8.02	–	10.83	1.57	11.66	UDP-glucosyl transferase 73C7
BnaA09g39260D	–	–	5.96	–	8.74	5.18	ACC synthase 1
BnaA09g29790D	1.00	1.43	–	3.60	–	3.30	UDP-glucosyl transferase 74B1
BnaA09g35660D	3.98	3.16	–	4.38	–	5.0	Transmembrane amino acid transporter family protein
BnaA09g40810D	–	–	2.68	1.46	–	–	DNA-binding protein phosphatase 1
BnaA09g34590D	–	–	0.99	1.02	–	–	Plant-specific GATA-type zinc finger transcription factor family protein
BnaA09g40670D	2.01	1.35	–	–	–	–	Protein of unknown function (DUF778)
BnaA09g34470D	2.13	1.85	–	–	–	–	tryptophan synthase alpha chain
BnaA09g31510D	–	–	4.07	2.55	3.90	3.26	AMP-dependent synthetase and ligase family protein

*RM is R-mock. The value in each comparison is a log2 (fold-change). R24 is resistant line 24 h post inoculation. S24 susceptible line 24 h post inoculation. R48 is resistant line 48 h post inoculation. S48 susceptible line 48 h post inoculation. R96 is resistant line 96 h post inoculation. S96 susceptible line 96 h post inoculation.*

## Discussion

### Classification and Functions of Identified Genes

The plant defense mechanism involves a wide range of responses in the recognition and handling of the disease (Wang et al., 2018), and a complicated network of genes was found to be expressed differentially in response to SSR within QTL regions. Different families of genes were upregulated in the R lines compared to the R mock and S lines.

One of the TIR-NBS-LRR class genes, *BnaC03g05380D*, present in the QTL region of *SRC3a*, was differentially expressed in R plants compared to R-mock plants at 48 hpi with a log_2_ fold-change (log_2_FC) of more than 1, and its expression was entirely absent in S plants at all time points with a log_2_FC of almost 10 at each time point. Although there were many genes of this family present in our QTL region, only this gene was found with high relative expression in R plants. The only function of NBS-LRR proteins reported so far is disease resistance, whereas its role in resistance is yet be confirmed for most of the genes. The simplest model suggested for NBS-LRR protein function is that they act as receptors and bind the effector molecules secreted by pathogens. Proteolysis either specific or general is predicted to have its role in controlling the amplitude of defense response and the magnitude of cell death related to hypersensitive response (McHale et al., 2006). TIR-NBS-LRR class genes on other chromosomes were also predicted in other studies to be involved in SSR resistance in *Brassica napus* (Wu et al., 2016a, b).

Two zinc-induced facilitator 1 genes that are close together in the C3 chromosome of the *SRC3a* QTL were highly upregulated in the R lines compared to the R-mock and S lines. *BnaC03g06010D* was upregulated in all comparisons at all time points, whereas *BnaC03g06020D* was upregulated at 48 hpi in both comparisons. The difference in expression between the R and S lines was more obvious than the difference between the R and R-mock lines. Zinc-induced facilitator 1 belongs to one of the largest families of transporters present in plants, the Major Facilitator Superfamily (MFS) (Pao et al., 1998). The functional significance of a great number of MFS transporters is yet not determined in the fundamental physiological processes (Remy et al., 2013). An important function of MFS transporters Arabidopsis ZIFL1 was observed in regulating polar auxin transport and drought tolerance (Remy et al., 2013). ZIFL genes in wheat were also characterized for their expression in response to Zn and Fe deficiency (Sharma et al., 2019).

*BnaC02g09450D*, a nitrilase 4 gene, was also highly upregulated in R lines at all three time points within all comparisons present in the QTL *SRC2a*. With reference to their role in plant-pathogen interactions, nitriles are involved in hormone synthesis, nutrient assimilation and detoxification of exogenous and endogenous nitriles (Howden and Preston, 2009). In plants, nitriles are involved in defense pathways and in mostly cases are linked with cyanide metabolism (Howden and Preston, 2009). Nitrile compounds are also involved in the metabolism of cyanogenic glycosides and glucosinolates and both molecules are recognized defense molecules in plants that provide protection against pathogen attack (Howden and Preston, 2009).

*BnaC02g48820D*, an EXORDIUM-like 2 gene in QTL *SRC2a*, was highly upregulated in R lines at 48 and 96 hpi compared to S and R-mock lines. While screening for potential mediators of brassinosteroid the EXORDIUM gene was identified as upregulated (Coll-Garcia et al., 2004). Brassinosteroid metabolism is known to be activated differentially and adopt changes in plants when encounter abiotic stresses or bacterial, fungal and viral pathogens via activation of different mechanisms (Bajguz and Hayat, 2009).

*BnaC02g00990D*, an ATAF2 subfamily of NAC proteins, was highly upregulated in R lines at all three time points of disease inoculation, with a more obvious difference being observed in comparison to S lines than compared to R-mock. These proteins can form multiple protein complexes and have the ability to regulate a number of cellular processes during plant development stages and in response to stress (Puranik et al., 2012). The ATAF subfamily transcription factors act as the convergence point for biotic and abiotic stress signaling (Mauch-Mani and Flors, 2009). ATAF2 function was identified as a repressor for the proteins related to pathogenesis in Arabidopsis (Delessert et al., 2005).

Two UDP-glucosyl transferase genes were upregulated at 24 hpi in both comparisons. *BnaA09g33440D*, a UDP-glucosyl transferase 73C7, was more than 8 log_2_FC at 24 hpi and was highly upregulated at 96 hpi. *BnaA09g29790D*, a UDP-glucosyl transferase 74B1, was more than 1 log_2_FC compared to both susceptible and resistant mock lines at 24 hpi present in the *SRA9a* QTL. The overexpression of *BnUGT74B1* in *B. napus* increased the aliphatic and indolic glucosinolates levels by 1.7 folds in leaves and in response to Sclerotinia infection showed less severe disease symptoms and tissue damage compared with the wild type control. UGT 73B3 and 73B5 responses to *Pseudomonas syringae* pv. *tomato* disease resistance have previously been reported and emphasize the importance of plant UGTs in plant-pathogen interactions (Langlois-Meurinne et al., 2005).

The other genes included in the QTL regions with upregulated expression (Table 5) in resistant lines at different time points are explained briefly in this study with their functions in disease resistance. The phylogenetic analysis in both distance-based and sequence-based analyses revealed an association of glycosyl hydrolase family 31 protein with defense enzymes (Opassiri et al., 2006). Aspartate is a precursor for the biosynthesis of several amino acids and derived metabolites that have unique and essential roles in plant development and defense response (de la Torre et al., 2014). Phosphoglycerate mutase-like family proteins are involved in glycolytic activity, which is essential for guard cell function in Arabidopsis (Zhao and Assmann, 2011). ARM repeat superfamily protein functions have been reported in protein sorting via ubiquitination, while the protein degradation plays an important role in achieving proteome plasticity in plants under environmental stress conditions to respond effectively (Sharma and Pandey, 2015). α/β-Hydrolase superfamily proteins are characterized to evolve complex and specialized chemical adaptations in response to varying biotic and abiotic conditions (Mindrebo et al., 2016). Plant *S*-adenosyl-L-methionine-dependent methyltransferases are the main enzymes in many metabolic pathways, including phenylpropanoid pathway (Joshi and Chiang, 1998), while reprogramming of the phenylpropanoid pathway is reported for SSR disease resistance in soybean (Ranjan et al., 2019). Gene repression via histone deacetylase plays a key regulatory role in plants to respond environmental stress (Luo et al., 2017). Polynucleotidyl transferase, a ribonuclease H-like superfamily protein, has an important role in mRNA deadenylation and mediation of stress responses (Walley et al., 2010). The ELMO/CED-12 family protein is required for the engulfment of dying cells and for cell migration (Gumienny et al., 2001). Many stresses are known to affect ribosomal protein gene transcript levels (McIntosh and Bonham-Smith, 2006). The roles of the KH-domain RNA-binding protein *At5g53060* of Arabidopsis is reported in jasmonic acid signaling and biotic-induced stress responses (Thatcher et al., 2015). Invertases have a role in the establishment of plant defense responses as during the infection pathogens use plant sugars for their own needs and force plants to alter their sugar content and trigger their defense responses (Tauzin and Giardina, 2014). Pyruvate kinase is a catalyst in the irreversible synthesis of pyruvate and ATP, which are both involved in multiple biochemical pathways (Baud et al., 2007). ACC synthase is reported to be involved in ethylene signaling in roots and responsible for immediate inhibition of root elongation in response to pathogen-associated molecular patterns (Tsang et al., 2011). Amino acid transporters are required for resource allocation processes during plant growth and development (Ortiz-Lopez et al., 2000). DNA-binding proteins from tobacco plants are identified as a novel class of plant-specific regulatory factors that are involved in plant-virus interactions (Castelló et al., 2011). The plant tryptophan biosynthetic pathway is involved in the production of many secondary metabolites with diverse functions (Zhang et al., 2008).

### Avoiding the Role of Flowering in the Identification of Resistance Genes

We have also observed that flowering time has an effect on the disease resistance trait in our study and in some past studies (Wei et al., 2014; Wu et al., 2019), but in this study, we only investigated the genes directly involved in resistance. Under natural conditions, it may be predicted that early flowering plants shed their petals earlier on leaf stem attachment and provide a long moist period for disease to develop before ripening compared to late flowering plants. However, late flowering plants might be involved in disease escape under natural conditions because they do not allow petals to stay on them for an extended period and take less time for ripening due to increased temperature. However, in artificial inoculation, we inoculated both genotypes at the same time when termination of flowering was completed in both genotypes, which may reduce the effect of the environment on disease development and emphasize genetic factors that are involved in disease resistance. There may be a difference in developmental stage at the time of artificial inoculation that might have its role in disease resistance, as susceptible lines have completed their flowering slightly earlier than resistant lines and are ahead in developmental stage in the course of ripening. There may be a more vigorous network of genes involved in different pathways that were activated in the resistant lines during the time of inoculation that might help the resistant lines cope better with disease. The colocalization of the QTLs involved in flowering and SSR resistance was also reported, and the genes that accelerate flowering time in plants are closely linked to genes that increase susceptibility to the disease (Wu et al., 2019). Common genetic regions are involved in flowering time and SSR resistance was also reported in other past studies (Wei et al., 2014). The early flowering with increased disease susceptibility was observed in our study which is consistent with the previous findings (Wei et al., 2014; Wu et al., 2019). There is a strong need to identify the pathways or mechanisms affecting flowering and SSR resistance at the same time, and emphasizing the study for genetic linkage between these two traits and how these traits may be used for improvement in cultivar development with increased resistance. We have attempted to avoid the role of flowering in our identification of disease resistance genes by the selection of extreme lines for resistance and susceptibility from the same population with almost the same flowering time. This approach helped us to identify DEGs in the same growth period of both lines that might be involved directly in resistance to SSR.

Our study supports the narrative that SSR resistance is a quantitative trait controlled by the complex network of genes, as stated earlier (Wu et al., 2013). Acquiring the complete resistance genes or network of genes involved in resistance remains a challenge. Identifying a resistant source with their regulatory mechanism is a complex task, especially in polyploid crops. Although there was no common QTL identified in all 3 years in this study, the correlation among the phenotypic measurements was high in all 3 years and most of the QTLs identified in this study are in accordance with previous findings (Zhao et al., 2006; Wu et al., 2013, 2016a, 2019; Wei et al., 2014, 2016). However, the environmental fluctuations like temperature, humidity and other factors may alter the plants adaptation in response to disease, especially for such a disease with a very complex pattern of pathogen–plant interaction. It was noted that both lines selected for RNA-seq initiated flowering more than 2 weeks later in 2019 compared to 2017 (Figure 5A). Considering the common QTLs in two seasons and with other studies, we sought to identify the genes within these QTL regions that contribute to SSR resistance. Comparative RNA-seq is a reliable technique that we can use to cover the gene expression analysis of all the genes within QTL regions. With the help of DEGs, we were able to identify some of the genes that make little but significant contributions to SSR resistance. The genes identified in this study have shown their relation to biotic or abiotic stress in previous studies, highlighting the importance of these genes and supporting our findings. One of the identified gene in our present study, *BnUGT74B1*, was reported earlier in an overexpression study to be directly involved in increasing glucosinolates content and increased resistance against *S. sclerotiorum* infection (Zhang et al., 2015). This consistence with the previously reported gene suggested that the identified genes may play important roles for development of resistant cultivars by introducing the reported genes via overexpression or with a breeding strategy to enhance their expression. We suggest that these genes identified in our studies may have a direct role in resistance and need to be studied more with their specific role to counter the fungal disease *S. sclerotiorum.* The detailed study of all the DEGs in resistant and susceptible lines with additional experiments will be discussed in a future article to understand the network of genes in the whole genome responsible for disease resistance.

## Data Availability Statement

The datasets presented in this study can be found in online repositories. The names of the repository/repositories and accession number(s) can be found: NCBI, SRA accession PRJNA616079.

## Author Contributions

YZ conceived the study. MQ and YZ designed the experiments. QZ, CF, and JW helped in data analysis. MS, RS, and SA helped in data collection. MQ and YZ wrote the manuscript. All the authors read and revised the manuscript.

## Conflict of Interest

The authors declare that the research was conducted in the absence of any commercial or financial relationships that could be construed as a potential conflict of interest.

## Footnotes

1 http://www.illumina.com/

2 http://statgen.ncsu.edu/qtlcart/WQTLCart.htm

3 ftp://ftp.ensembl.org/pub/release-94/gtf/mus_musculus/
