# Supplementary material for: Identification of QTLs Containing Resistance Genes for Sclerotinia Stem Rot in Brassica napus Using Comparative Transcriptomic Studies
Source: Front Plant Sci. 2020 Jun 10;11:776. doi: 10.3389/fpls.2020.00776 (PMC7325899; doi:10.3389/fpls.2020.00776)
Supplement: Supplementary file 2 [file Image_1.pdf]

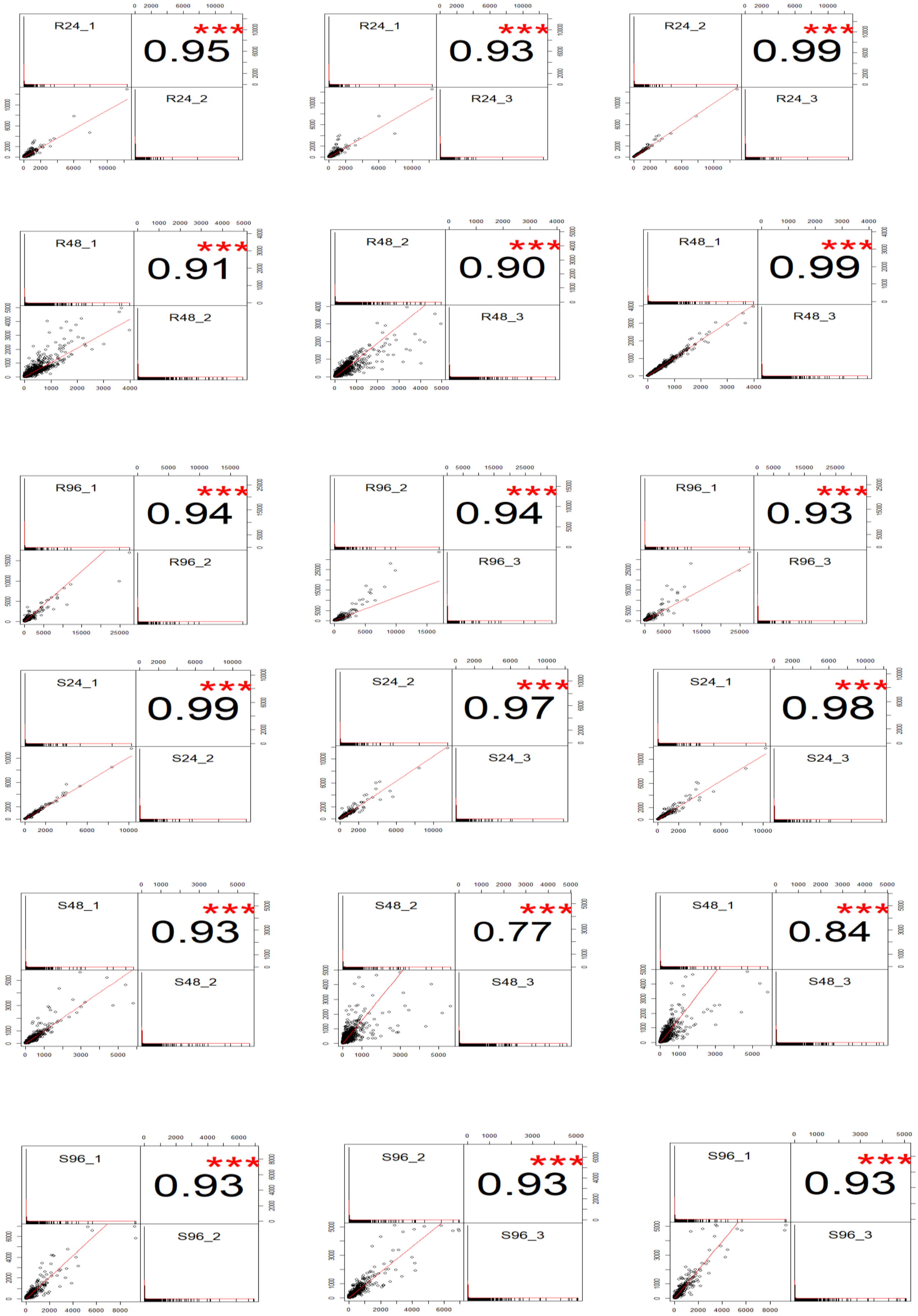
R24_1
*** 0.95
R24_2
R24_1
*** 0.93
R24_3
R24_2
*** 0.99
R24_3
R48_1
*** 0.91
R48_2
R48_2
*** 0.90
R48_3
R48_1
*** 0.99
R48_3
R96_1
*** 0.94
R96_2
R96_2
*** 0.94
R96_3
R96_1
*** 0.93
R96_3
S24_1
*** 0.99
S24_2
S24_2
*** 0.97
S24_3
S24_1
*** 0.98
S24_3
S48_1
*** 0.93
S48_2
S48_2
*** 0.77
S48_3
S48_1
*** 0.84
S48_3
S96_1
*** 0.93
S96_2
S96_2
*** 0.93
S96_3
S96_1
*** 0.93
S96_3

Supplementary figure 1. Correlation analysis among the replications used in RNA-seq analysis at each time point of resistant and susceptible lines.
